# Resistance Training in Type 2 Diabetic Patients Improves Uric Acid levels

**DOI:** 10.2478/hukin-2014-0085

**Published:** 2014-11-12

**Authors:** Moisés S.S.R. Sousa, Francisco J.F. Saavedra, Gabriel R. Neto, Giovanni S. Novaes, Antonio C. R. Souza, Verônica P. Salerno, Jefferson S. Novaes

**Affiliations:** 1 University of Trás-os-Montes and Alto Douro (UTAD); Doctoral Program in Sport Science – PORTUGAL.; 2 State University of Pará (UEPA), Belém, Pará – BRAZIL.; 3 Federal University of Rio de Janeiro (UFRJ). Physical Education - Graduate Program. Rio de Janeiro, RJ – BRAZIL.

**Keywords:** muscular strength, uric acid, diabetes, resistance training

## Abstract

Resistance training (RT) can provide several benefits for individuals with Type 2 diabetes. The aim of this study was to investigate the effects of resistance training on the strength levels and uric acid (UA) concentration in individuals with Type 2 diabetes. The study included 68 patients (57.7±9.0 years) that participated in an organized program of RT for 12 weeks. The volunteers were divided into two groups: an experimental group (EG; n=34) that performed the resistance training program consisting of seven exercises executed in an alternating order based on segments; and a control group (CG; n=34) that maintained their normal daily life activities. Muscle strength and uric acid were measured both pre- and post-experiment. The results showed a significant increase in strength of the subjects in the EG for all exercises included in the study (p<0.001). Comparing the strength levels of the post-test, intergroup differences were found in supine sitting (p<0.001), leg extension (p<0.001), shoulder press (p<0.001), leg curl (p=0.001), seated row (p<0.001), leg press (p=0.001) and high pulley (p<0.001). The measured uric acid was significantly increased in both experimental and control groups (p<0.001 and p=0.001, respectively). The intergroup comparison showed a significant increase for the EG (p=0.024). We conclude that the training program was effective for strength gains despite an increase in uric acid in Type 2 diabetics.

## Introduction

Diabetes mellitus (DM) is related to a group of metabolic diseases characterized by a chronic hyperglycemia attributed to an inadequate secretion or diminished action of insulin (Kommoju and Reddy, 2011). Increased physical activity is a lifestyle strategy that can improve glucose levels and should be performed soon after diagnosis of Type 2 diabetes, independent of weight loss, reduced energy intake, monitoring of portions of carbohydrates and limited saturated fat intake (Franz et al., 2002).

The American College of Sports Medicine (ACSM) and the American Diabetes Association (ADA) advocate physical activity as a treatment method for individuals with DM. Currently, a total energy expenditure of at least 1000 kcal/week or 150 minutes per week of aerobic exercise of moderate intensity or 90 minutes a week of vigorous aerobic exercise is recommended (ACSM, 2010b; Sigal et al., 2004).

When comparing different physical activities practiced by individuals with DM, resistance training (RT) provides a greater advantage than aerobic exercise in relation to strength and muscle mass gain (Hunter et al., 2004; Kraemer et al., 2002). The loss of muscle mass is a characteristic of diabetes (Zabaglia et al., 2009), and RT can be an important exercise for diabetic individuals since it promotes an increase in lean muscle mass, muscle strength, the basal metabolic rate and sensitivity to insulin, which is reflected as an improved control of blood glucose levels (Ciolac and Guimarães, 2004).

In addition, other important blood markers that can serve as biomarkers for impaired physical performance are changed with the practice of RT such as uric acid (UA), urea and ammonia as well as the activity of enzymes such as creatine kinase. It has been suggested that plasma UA levels in particular can be a marker for monitoring the effects of RT because of its association with the catabolic state and its increase can serve as an indicator of a decrease in muscle protein (Cunha et al., 2006), in addition to being associated with type 2 diabetics and hypertension in overweight subjects (Nakanishi et al., 2003).

Considering that Type 2 diabetics leads to an alteration in glucose metabolism, exercise can improve glucose uptake by skeletal muscles and RT can promote increased muscle strength. Thus, we hypothesized that regular RT could be used as an additional tool to control the detrimental effects of diabetes. When verifying the pertinent literature, there was a knowledge gap regarding the chronic responses of a resistance training program on the levels of uric acid in type 2 diabetics. Therefore, the aim of this study was to analyze the effects of a 12-week RT program on muscle strength and plasma UA levels in patients with Type 2 diabetes.

## Material and Methods

### Participants

Prior to recruiting participants, this study was approved by the Ethics Committee in Research of the State University of Pará (UEPA; Assent number 0013.0.412000-08) confirming that all procedures complied with the ethical committee standards on human experimentation developed in accordance to the Helsinki Declaration of 1975 as amended in 2000. Participants were chosen at random from a group of 340 patients enrolled in the program for cardiovascular prevention at the Casa do Diabético in Belém do Pará, Brazil and were registered as being sedentary. Individuals were excluded based on musculoskeletal, mental, neurological and hormonal disorders. After the initial assessment, 68 patients (57.7±9.0 years) of both sexes, sedentary and with diabetes mellitus Type 2, were included in the study. Each signed a consent form authorizing participation in the research after receiving full disclosure of the design of the study. In addition, each participant declared that they had not participated in regular exercise in the six months prior to the start of the program. The study consisted of 12 weeks of RT and all evaluations were performed before and after training. The volunteers were divided into two groups: an experimental group (EG; n=34) and a control group (CG; n=34). The study can be characterized as quasi-experimental (Sousa et al., 2007).

### Anthropometric assessment

Body mass (BM) was measured on a balance platform (Fillizola®; São Paulo, SP, Brazil), with an accuracy of 0.1 kg. Body height (BH) was determined with a stadiometer (Sanny, model ES 2020, American Medical do Brazil Ltda.) with an accuracy of 0.01 mm. The body mass index (BMI) was calculated from the standards established by the ACSM (ACSM, 2010a).

### Blood samples

Fasting blood samples were collected for uric acid analysis following the recommendation of Sacks et al. (2011). Venous blood samples were obtained by puncture of the cubital or basilic vein using a disposable hypodermic syringe. The blood was transferred into tubes containing EDTA and immediately centrifuged for 15 minutes (3000 rpm @ 4 °C) to separate plasma. Plasma was collected and stored at −80 °C for later analysis. Blood was collected before and after 12 weeks of resistance training.

### Uric acid assessment

The analysis of uric acid was performed by the automated enzymatic method using the device (Cobas Mira, Roche) at Beneficente Portuguesa Hospital, through a urease enzyme based assay using a commercial kit (Roche) according to the manufacturer’s instructions.

### Muscular strength assessments

A 12RM test was used to predict the maximum load for each subject considering their lack of experience, training and a sedentary life style. The 12 RM testing protocol consisted of: (a) a warm up of 40–60% of the maximum perceived effort to allow 12 RM; (b) five maximum repetitions, after a 1 min rest at 60–80% of the maximum perceived effort to allow 12 RM; (c) the load test was started after 1 min rest and each subject performed a maximum of three attempts for each exercise with an interval of 5 min for each attempt; d) recording of the last complete execution was determined by the moment when the subject lost the ability to perform the movement correctly and the test was interrupted. The recorded maximum load for 12 repetitions reflected the last execution of the full movement before the concentric muscular failure. A limiting amplitude motion was used to determine the start and end positions of each exercise as described by Kaminsky (2006).

All participants underwent load testing and performed training exercises in alternating order based on body segment and in the following order: supine sitting (SS), leg extension (LE), shoulder press (SP), leg curl (LC), seated row (SR), leg press (LP) and high pulley (HP). To reduce the margin of error in the tests, the following strategies were adopted: (a) familiarization with the exercise and equipment before the test, what made the subject aware of the collection of routine data; (b) instructions on the proper techniques of performing the exercises; (c) careful attention by the evaluator for the position adopted by the practitioner; d) use of verbal stimuli; e) confirmation of weights on precision scales. All measurements were performed before and after training. The assessment and training were carried out at the Laboratório de Esforço Resistido (LERES) at Universidade Estadual do Pará.

### Resistance Training

Participants performed the prescribed exercises 3 times per week for 12 weeks. Overall, the RT was composed of seven exercises that were conducted with three sets of 12 RM with a two-minute interval between exercises and a minimum of 48 hours rest between the days of RT. An adaptation phase of four weeks was implemented and composed of four exercises: SS, LE, LC and SP. After this period, three exercises were added: SR, LP, HP. The progression of the load occurred with addition of 5 to 10% when it reached 15-RM with appropriate technique. At all times, evaluators monitored subjects for proper technique and limited exercises based on the perception of pain or discomfort by the participant.

### Statistical analysis

Initially, calculations were performed using the Kolmogorov-Smirnov test and Bartlett criterion, to show that the variables demonstrated a normal distribution and homoscedasticity. Subsequently, the Wilcoxon test was used for intragroup comparison and the Mann-Whitney test for intergroup comparison. All statistical analyzes were performed using SPSS (version 20.0, SPSS Inc., Chicago, IL). The level of significance for all analyzes was set at p<0.05. The effect size was used to determine the magnitude of changes in the groups (difference between the scores of the post-test and pre-test standard deviation divided by the pre-test) as established by Rhea (2004).

## Results

Table 1 lists the multiple characteristics of the participants including their age distribution, anthropometric measurements, physical performance and their UA levels. An analysis of the age, body mass, body height and BMI showed that the subjects had a normal distribution pattern (p>0.05). However, the variables of physical performance and UA concentration did not display a normal distribution (p<0.05).

Table 2 shows the intra- and intergroup comparison. Through the intragroup comparison, the EG group showed a significant increase in strength after training (post-test) for all exercises performed (p<0.001). The intergroup, post-test comparison between the groups EG and CG showed a significant difference in the exercises SS (p<0.001), LE (p<0.001), SP (p<0.001), LC (p=0.001), SR (p<0.001), LP (p=0.001), and HP (p<0.001).

**Figure 1:**
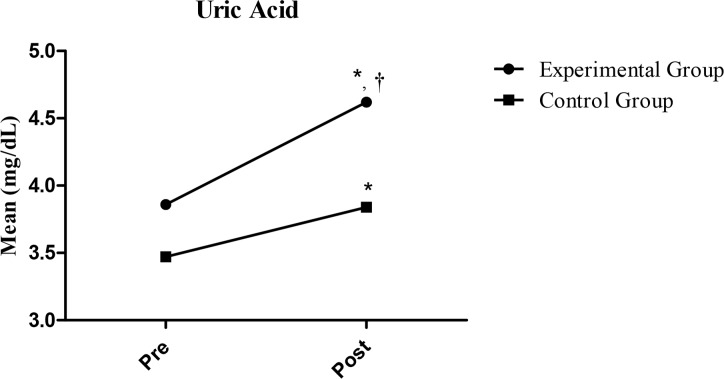
Comparative analysis of uric acid concentration for the experimental group and control group before and after the study. ^*^ Intragroup significant difference (p<0.05) compared between pre and post. ^†^ Intergroup significant difference (p<0.05) compared between EG-post and CG-post (p<0.05).

Figure 1 displays the plasma uric acid concentration of the two groups, EG and CG, measured at the start (pre-test) and after the study (post-test, 12 weeks). The intragroup comparisons showed a significant increase in the UA concentration within both groups (p<0.001 and p=0.001, respectively). An intergroup comparison between the EG and CG post-test measurements showed that the concentration of UA was greater in the EG, but not significantly (p=0.024).

## Discussion

The present study investigated the effects of 12 weeks of resistance training (RT) on muscle strength and uric acid concentration in the plasma of Type 2 diabetes subjects. Our results showed that the experimental group (EG) had higher scores in the variables of strength and UA concentration in comparison to the control group (CG). To our knowledge, this is the first study to show the significant influence of resistance training on UA levels.

A significant increase of moderate magnitude in strength was observed in all exercises of the EG (SS, LE, SR, LP, HP), suggesting that the improvement was a result of the specific RT in the experimental protocol consisting of different exercises for the upper and lower limbs. It is known that RT promotes an incremental increase in the number of motor units and, at the same time, improves synchronization and firing frequency of motor units through neural adaptations (Castaneda et al., 2002; Hameed et al., 2012). All of these could contribute to the gain in muscle strength demonstrated in Type 2 diabetic subjects in this study and is consistent with studies on muscle strength in type 2 diabetic individuals after a program of resistance exercises (Andersen et al., 2004; Castaneda et al., 2002; Hameed et al., 2012). The results of our study support the hypothesis that RT is an effective intervention strategy to prevent muscle weakness of diabetics. Our findings also showed that the experimental group improved levels of strength with every exercise, regardless of the anatomical location of the muscle groups worked.

Aspects of RT utilized in our study that differed from previous reports include the methods for more precisely determining maximum load and safety measures. In a study with untrained individuals with Type 2 diabetes that performed progressive RT over eight weeks, muscle strength was evaluated on the basis of a prediction test of 1 RM on the bench and leg press (Hameed et al., 2012). While a significant improvement in strength was observed along with improved blood glucose levels, the precision of the strength tests was limited by the ability to evaluate the load in the individuals tested. In this study, the maximum load was determined by an indirect test of 1 RM, which was included in the high intensity RT protocol. This load was verified by a 12 RM test that was used as the basis for the training protocol. Performing the exercises with a 12 to 15 RM is more characteristic of a training protocol aimed at muscle endurance (Kraemer et al., 2002), which could provide long term benefits to the participants. Another benefit of the 12 RM test is preventing from possible lesions in muscle caused by maximum loads, a major safety concern for diabetics. Safety was further improved by an intervention program that was included during the performance of the RT exercises. Overall, the present study implemented an RT program with various exercises that included tests for each subject at a high level of safety, which is an improvement when compared with previous studies that involved fewer exercises without load tests for all and, in some cases, utilized improper tests for this population.

The concentration of UA has been suggested as a marker for the impact of an RT session since the concentration of nitrogen that residues in the plasma can be an indicator of a decrease in muscle proteins associated with the catabolic state (Cunha et al., 2006). Uric acid plays an important and complex role in oxidative and anti-oxidative processes that has been reported to be positively correlated with cardiovascular disease and various measures of psychosocial stress (Bove et al., 2010), especially in high risk patients (Tsouli et al., 2006). In overweight people, Type 2 diabetics and hypertension are associated with the concentrations of uric acid (Nakanishi et al., 2003). Also in Type 2 diabetes, higher levels of UA were associated with many components of the metabolic syndrome and patients with a metabolic syndrome displayed higher levels of UA (Salehidoost et al., 2012). However, when longitudinal changes were concerned, the concentration of UA was not related to MS (Oda, 2013).

We measured UA values that were significantly higher by an intragroup comparison for the EG, which can be best explained by the fact that RT accelerates the metabolic process and stimulates the production of UA (Urhausen and Kindermann, 2002). Waring et al. (2003) claimed that high UA concentrations were associated with increased serum antioxidant capacity and reduced oxidative stress during acute physical exercise in healthy subjects. Part of the increase in UA levels can be attributed to the higher BMI measured in the EG, which corroborates with increases in uric acids in overweight subjects (Nakanishi et al., 2003). However, we also recorded a higher UA concentration in the CG at the end of the 12-week study that would be normalized during the statistical analysis for significance. The increase in UA observed in the CG could be caused by the consumption of food in all participants as shown in the study of Gleeson (2002). Attempts were made to minimize this possible variable by instructing each group to follow the guidance of the food program for the prevention of cardiovascular diseases of the Casa do Diabetico at Belém do Pará.

Importantly, UA is not an independent factor for predicting the metabolic syndrome (Salehidoost et al., 2012) and neither can be used as the sole factor in monitoring a training session (Gleeson, 2002; Kindermann, 1986; Urhausen et al., 1998). It is necessary to control other biochemical markers during a program of RT such as the adrenocorticotropic hormone, growth hormone, cortisol and catecholamines (Urhausen and Kindermann, 2002). The fact that we have not collected these biochemical markers in our study can be considered a limiting factor for the extrapolation of our results. Overall, our findings are of interest since there was a difference between the study groups that suggests that RT has benefits beyond muscle strengthening and can be a tool in combating oxidative stress in type 2 diabetes by increasing UA levels.

## Conclusion

A twelve week resistance training protocol increased muscle strength in all muscle groups engaged despite higher UA concentrations observed in Type 2 diabetics. The increment in strength measured can improve the health status in the population with Type 2 diabetes by reducing the risks associated with this disease. We may conclude that RT is an excellent tool for the control and maintenance of the quality of life of people suffering from Type 2 diabetes.

## Figures and Tables

**Table 1:** Characteristics of the participants

Variable	**Experimental Group (EG=34)**		**Control Group (CG=34)**	

	Mean ± SD	K-S	Mean ± SD	K-S
Age (years)	58.94 ± 10.65	0.200	56.52 ± 7.05	0.200
Body mass (kg)	71.61 ± 11.84	0.200	72.67 ± 10.17	0.200
Body height (m)	1.55 ± 0.08	0.127	1.61 ± 0.07	0.200
BMI (kg/m^2^)	29.64 ± 4.26	0.064	27.75 ± 3.19	0.200
SS (kg)	8.58 ± 4.91	0.000	5.67 ± 5.06	0.000
LE (kg)	11.91 ± 5.36	0.000	14.41 ± 5.47	0.003
SP (kg)	1.94 ± 3.63	0.000	2.26 ± 2.26	0.000
LC (kg)	3.97 ± 3.2	0.000	5.50 ± 5.22	0.000
SR (kg)	22.02 ± 8.63	0.000	22.05 ± 8.97	0.052
LP (kg)	21.17 ± 9.29	0.000	26.76 ± 12.78	0.000
HP (kg)	14.44 ± 4.83	0.000	16.32 ± 17.85	0.000
UA (mg/dL)	3.86 ± 1.19	0.047	3.47 ± 1.28	0.018

SD = standard deviation; BMI = body mass index; SS = supine sitting; LE = leg extension; SP = shoulder press; LC = leg curl; SR = seated row; LP = leg press; HP = high pulley; UA = uric acid; K-S = Kolmogorov-Smirnov.

**Table 2:** Muscle strength comparison Intra and Intergroup for each exercise

Variable	Experimental Group (EG=34)				Control Group (CG=34)			

	Pre-test	Post-test	p	ME	Pre-test	Post-test	p	ME
SS (kg)	8.58±4.91	14.85±6.74	0.001*†	1.27 Moderate	5.67±5.06	5.44±5.62	0.285	−0.04 Trivial
LE (kg)	11.91±5.36	22.35±7.51	0.001*†	1.94 Moderate	14.41±5.47	14.11±6.21	0.684	−0.05 Trivial
SP (kg)	1.94±3.63	6.0±3.61	0.001*†	1.11 Small	2.26±2.26	2.02±2.52	0.285	−0.10 Trivial
LC (kg)	3.97±3.2	7.70±4.37	0.001*†	1.16 Small	5.50±5.22	4.55±4.66	0.102	−0.18 Trivial
SR (kg)	22.02±8.63	38.11±12.55	0.001*†	1.86 Moderate	22.05±8.97	22.79±10.31	0.564	0.08 Trivial
LP (kg)	21.17±9.29	35.11±13.72	0.001*†	1.50 Moderate	26.76±12.78	25.88±11.31	0.251	−0.06 Trivial
HP (kg)	14.44±4.83	23.20±7.9	0.001*†	1.81 Moderate	16.32±17.85	14.35±7.13	0.323	−0.11 Trivial

SS = supine sitting; LE = leg extension; SP = shoulder press; LC = leg curl; SR = seated row; LP = leg press; HP = high pulley; ME = magnitude of effect.

* Significant difference between pre- and post-test (p<0.05);

† Significant difference between EG and CG in the post-test (p<0.05).

## References

[b1-jhk-43-17] ACSM. American College Sports Medicine (2010a). ACSM guidelines for stress testing and prescription.

[b2-jhk-43-17] ACSM (2010b). Exercise and Type 2 diabetes: American College of Sports Medicine and the American Diabetes Association: joint position statement. Exercise and Type 2 diabetes. Med Sci Sports Exerc.

[b3-jhk-43-17] Andersen H, Nielsen S, Mogensen CE, Jakobsen J (2004). Muscle strength in Type 2 diabetes. Diabetes.

[b4-jhk-43-17] Bove M, Carnevali L, Cicero AF, Grandi E, Gaddoni M, Noera G, Gaddi AV (2010). Psychosocial factors and metabolic parameters: Is there any association in elderly people? The Massa Lombarda Project. Aging Ment Health.

[b5-jhk-43-17] Castaneda C, Layne JE, Munoz-Orians L, Gordon PL, Walsmith J, Foldvari M, Roubenoff R, Tucker KL, Nelson ME (2002). A randomized controlled trial of resistance exercise training to improve glycemic control in older adults with Type 2 diabetes. Diabetes Care.

[b6-jhk-43-17] Ciolac EG, Guimarães GV (2004). Physical exercise and metabolic syndrome. Rev Bras Med Esporte.

[b7-jhk-43-17] Cunha GS, Ribeiro JL, Oliveira AR (2006). Overtraining: theories, diagnosis and markers. Rev Bras Med Esporte.

[b8-jhk-43-17] Franz MJ, Bantle JP, Beebe CA, Brunzell JD, Chiasson JL, Garg A, Holzmeister LA, Hoogwerf B, Mayer-Davis E, Mooradian AD (2002). Evidence-based nutrition principles and recommendations for the treatment and prevention of diabetes and related complications. Diabetes Care.

[b9-jhk-43-17] Gleeson M (2002). Biochemical and immunological markers of overtraining. J Sports Sci Med.

[b10-jhk-43-17] Hameed UA, Manzar D, Raza S, Shareef MY, Hussain ME (2012). Resistance training leads to clinically meaningful improvements in control of glycemia and muscular strength in untrained middle-aged patients with type 2 diabetes mellitus. N Am J Med Sci.

[b11-jhk-43-17] Hunter GR, McCarthy JP, Bamman MM (2004). Effects of resistance training on older adults. Sports Med.

[b12-jhk-43-17] Kaminsky LA (2006). ACSM’s resource manual for Guidelines for exercise testing and prescription.

[b13-jhk-43-17] Kindermann W (1986). Overtraining–an expression of faulty regulated development. translated from. Deutsche Zietschrift Fur Sportmedizin.

[b14-jhk-43-17] Kommoju UJ, Reddy BM (2011). Genetic etiology of Type 2 diabetes mellitus: a review. Int J Diabetes Dev Ctries.

[b15-jhk-43-17] Kraemer WJ, Adams K, Cafarelli E, Dudley GA, Dooly C, Feigenbaum MS, Fleck SJ, Franklin B, Fry AC, Hoffman JR (2002). American College of Sports Medicine position stand. Progression models in resistance training for healthy adults. Med Sci Sports Exerc.

[b16-jhk-43-17] Nakanishi N, Okamoto M, Yoshida H, Matsuo Y, Suzuki K, Tatara K (2003). Serum uric acid and risk for development of hypertension and impaired fasting glucose or Type II diabetes in Japanese male office workers. Eur J Epidemiol.

[b17-jhk-43-17] Oda E (2013). Serum uric acid is an independent predictor of metabolic syndrome in a Japanese health screening population. Heart and vessels.

[b18-jhk-43-17] Rhea RM (2004). Determining the magnitude of treatment effects in strength training research through the use the effect size. J Strength Cond Res.

[b19-jhk-43-17] Sacks DB, Arnold M, Bakris GL, Bruns DE, Horvath AR, Kirkman MS, Lernmark A, Metzger BE, Nathan DM (2011). Position statement executive summary: guidelines and recommendations for laboratory analysis in the diagnosis and management of diabetes mellitus. Diabetes Care.

[b20-jhk-43-17] Salehidoost R, Aminorroayai A, Zare M, Amini M (2012). Is uric acid an indicator of metabolic syndrome in the first-degree relatives of patients with Type 2 diabetes?. J Res Med Sci.

[b21-jhk-43-17] Sigal RJ, Kenny GP, Wasserman DH, Castaneda-Sceppa C (2004). Physical activity/exercise and type 2 diabetes. Diabetes Care.

[b22-jhk-43-17] Sousa VD, Driessnack M, Mendes IAC (2007). An overview of research designs relevant to nursing: Part 1: quantitative research designs. Rev Latino-Am Enfermagem.

[b23-jhk-43-17] Tsouli SG, Liberopoulos EN, Mikhailidis DP, Athyros VG, Elisaf MS (2006). Elevated serum uric acid levels in metabolic syndrome: An active component or an innocent bystander?. Metabolism.

[b24-jhk-43-17] Urhausen A, Gabriel HH, Kindermann W (1998). Impaired pituitary hormonal response to exhaustive exercise in overtrained endurance athletes. Med Sci Sports Exerc.

[b25-jhk-43-17] Urhausen A, Kindermann W (2002). Diagnosis of Overtraining: What Tools Do We Have?. Sports Med.

[b26-jhk-43-17] Zabaglia R, Oliveira AC, Urtado CB, Souza TMF (2009). Effect of resistance training in people with diabetes mellitus. Rev Bras Prescr Fisiol Exerc.

[b27-jhk-43-17] Waring WS, Convery A, Mishra V, Shenkin A, Webb DJ, Maxwell SRJ (2003). Uric acid reduces exercise-induced oxidative stress in healthy adults. Clin Sci.

